# Red cell distribution width predicts cardiovascular complications after high-risk surgery

**DOI:** 10.1186/cc12170

**Published:** 2013-03-19

**Authors:** M Geisen, HD Aya, C Ebm, MA Hamilton, J Ball, M Grounds, A Rhodes, M Cecconi

**Affiliations:** 1 St George's Healthcare NHS Trust, London, UK

## Introduction

The red-cell distribution width (RDW) is associated with cardiovascular morbidity and mortality and is a predictor of ICU survival. It was the aim of this study to investigate the potential of RDW to predict postoperative cardiovascular complications (new onset of treated arrhythmias, myocardial ischaemia or initiation of vasopressor support) and its association with markers of tissue perfusion (serum lactate >2.5 mmol/l).

## Methods

Analysis of prospectively recorded data for a register of patients admitted to an 18-bed ICU in a large teaching hospital after undergoing high-risk surgery. Haemodynamic and laboratory parameters during the first 12 hours of ICU stay were recorded as well as demographic characteristics. Assessment for postoperative complications was performed using the postoperative morbidity survey and the Clavien-Dindo classification. In addition, clinical outcome data (hospital mortality, length of ICU stay, length of hospital stay, readmission to the ICU) were recorded.

## Results

**Figure 1:**
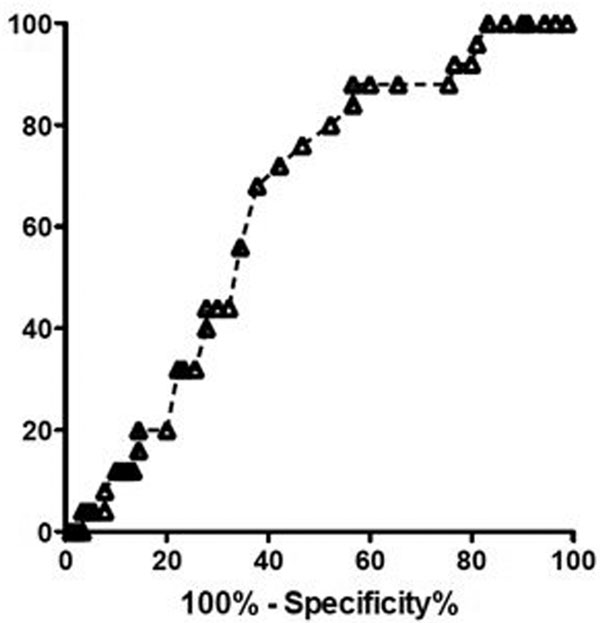
**Prediction of hyperlactataemia according to RDW**.

A total of 119 patients were included. Seventy-six (63%) patients developed complications postoperatively. Thirty patients (25.2%) developed cardiovascular complications. These patients had a higher median RDW than patients without cardiovascular complications (14.7 vs. 13.8%, *P <*0.05). RDW on ICU admission was associated with serum lactate concentration on ICU admission: receiver operating characteristic analysis showed an area under the curve of 0.68 (SE = 0.06, *P *= 0.005; see Figure 1). RDW >14.35% predicted hyperlactataemia with a sensitivity of 76.0% and a specificity of 71.1%.

## Conclusion

RDW is a potential parameter for perioperative risk stratification. Further studies will have to elucidate the ability of acute elevations in RDW to reflect impaired tissue perfusion.
